# Additive Benefits of Radium-223 Dichloride and Bortezomib Combination in a Systemic Multiple Myeloma Mouse Model

**DOI:** 10.3390/ijms22115570

**Published:** 2021-05-25

**Authors:** Mari I. Suominen, Jenni Mäki-Jouppila, Anna Huhtinen, Birgitta Sjöholm, Jukka P. Rissanen, Anniina Luostarinen, Katja M. Fagerlund, Esa Alhoniemi, Gerhard Siemeister, Dominik Mumberg, Sanna-Maria Käkönen, Arne Scholz

**Affiliations:** 1 Pharmatest Services Oy, 20520 Turku, Finland; mari.suominen@pharmatest.com (M.I.S.); jenni.maki-jouppila@pharmatest.com (J.M.-J.); jukka.rissanen@pharmatest.com (J.P.R.); anniina.luostarinen@pharmatest.com (A.L.); katja.fagerlund@pharmatest.com (K.M.F.); 2 Aurexel Life Sciences Oy, 21240 Askainen, Finland; anna.huhtinen@aurexel.com (A.H.); birgitta.sjoholm@aurexel.com (B.S.); sanna.kakonen@aurexel.com (S.-M.K.); 3 Vincit Oyj, 20500 Turku, Finland; esa.alhoniemi@vincit.fi; 4 Nuvisan-ICB GmbH, Therapeutic Oncology Research, 13353 Berlin, Germany; gerhard.siemeister@nuvisan.com; 5 Bayer AG, Research & Development, Pharmaceuticals, 13353 Berlin, Germany; dominik.mumberg@bayer.com; 6 Department of Cell Biology and Anatomy, Faculty of Medicine, University of Turku, 20014 Turku, Finland

**Keywords:** multiple myeloma, bortezomib, radium-223, dexamethasone, myeloma bone disease, osteoblast, osteoclast, targeted alpha-therapy, 5TGM1 mouse model, systemic model

## Abstract

Osteolytic bone disease is a hallmark of multiple myeloma (MM) mediated by MM cell proliferation, increased osteoclast activity, and suppressed osteoblast function. The proteasome inhibitor bortezomib targets MM cells and improves bone health in MM patients. Radium-223 dichloride (radium-223), the first targeted alpha therapy approved, specifically targets bone metastases, where it disrupts the activity of both tumor cells and tumor-supporting bone cells in mouse models of breast and prostate cancer bone metastasis. We hypothesized that radium-223 and bortezomib combination treatment would have additive effects on MM. In vitro experiments revealed that the combination treatment inhibited MM cell proliferation and demonstrated additive efficacy. In the systemic, syngeneic 5TGM1 mouse MM model, both bortezomib and radium-223 decreased the osteolytic lesion area, and their combination was more effective than either monotherapy alone. Bortezomib decreased the number of osteoclasts at the tumor–bone interface, and the combination therapy resulted in almost complete eradication of osteoclasts. Furthermore, the combination therapy improved the incorporation of radium-223 into MM-bearing bone. Importantly, the combination therapy decreased tumor burden and restored body weights in MM mice. These results suggest that the combination of radium-223 with bortezomib could constitute a novel, effective therapy for MM and, in particular, myeloma bone disease.

## 1. Introduction

Multiple myeloma (MM) is the second most common hematological malignancy [1]. It is the most frequent malignancy to affect bone, with up to 80% of patients developing bone disease characterized by destructive bone lesions and an increased risk for skeletal fractures associated with increased morbidity and mortality [2,3,4]. MM cells produce paracrine factors that stimulate osteoclast-mediated bone resorption and suppress osteoblast function, resulting in osteolytic bone lesions and reduced bone formation [5]. Although major advances have been made during recent years, the disease remains incurable.

Proteasome inhibition was identified as a target for MM treatment in the early 2000s. Bortezomib, the first-in-class approved drug, is now widely used, both as a first-line treatment for newly diagnosed MM and in patients with relapsed or refractory MM. Bortezomib is used either as a single agent or in combination treatment, for instance with immunomodulatory agents, steroids, or conventional chemotherapy drugs [6,7,8]. Besides their direct antiproliferative and cytotoxic effects in MM cells, bortezomib and other proteasome inhibitors have been shown to restore the impaired osteoblast activity typically observed in MM [9,10,11].

Radium-223 dichloride (radium-223, Xofigo) is an alpha particle emitter and a calcium-mimetic that selectively binds to the hydroxyapatite in bone and preferentially targets the areas of increased bone turnover, such as bone metastases. It is the first targeted alpha therapy (TAT) developed, and it has been approved by the US Food and Drug Administration (FDA) and the European Medicines Agency (EMA) for castration-resistant prostate cancer patients with symptomatic bone metastases but no known visceral metastases [12,13,14,15]. It has been shown to improve overall survival and to delay symptomatic skeletal complications [12,14]. Radium-223 uptake and selectivity are mediated by both an active and a passive component. Osteoblasts actively incorporate radium-223 into the bone matrix [16], and the degree of osteoblastic activity correlates with the level of radium-223 incorporation. In addition to active osteoblast-mediated incorporation, passive binding of radium-223 to hydroxyapatite via anion exchange against calcium may contribute to the uptake [16]. We have previously demonstrated that radium-223 possesses a dual mode of action in prostate cancer bone metastasis mouse models: it inhibits tumor growth and suppresses tumor-induced pathological bone formation, which are both processes involved in the vicious cycle of osteoblastic bone metastases. At the cellular level, radium-223, deposited into the intralesional area and the surrounding bone matrix, was shown to induce difficult-to-repair, potentially cytotoxic DNA double-strand breaks (DSBs) in tumor cells, osteoblasts, and osteoclasts located within the 80–100 µm range of alpha emitters [15,17].

Apart from prostate cancer, only a limited number of studies have reported the use of radium-223 in other malignancies affecting bone [12,13]. However, investigating such cancers, and in particular cancer-induced bone disease, and understanding their effects and modes of action, could provide more treatment options in a wider setting of malignant bone diseases. For example, in a mouse model of osteolytic breast cancer bone metastasis, radium-223 induced DSBs in tumor cells, decreased the number of osteoclasts, reduced the development of osteolytic lesions, and ultimately improved survival [18]. The first clinical signals support the relevance of these preclinical findings also for breast cancer patients [19,20,21]. Several clinical trials are currently ongoing to determine the efficacy and safety of radium-223 in combination with other agents like the androgen inhibitor enzalutamide (NCT02194842/PEACE III, NCT04237584/ESCALATE), checkpoint inhibitors (NCT03996473, NCT04109729), DNA damage response inhibitors (NCT03317392, NCT04071236), and chemotherapeutics (NCT03574571/DORA) in different tumor types.

In this study, we investigated the effect of radium-223, bortezomib, and their combination on myeloma cell proliferation in vitro and in the syngeneic 5TGM1 mouse MM model in vivo. This model recreates features of active human MM, including the secretion of the IgG2b monoclonal paraprotein as well as the pronounced osteolytic lesions in bone [22,23,24,25].

## 2. Results

### 2.1. Radium-223 Inhibits the Proliferation of Various MM Cell Lines and Shows Additive Activity in Combination with Bortezomib in 5TGM1 Cells In Vitro

**Figure 1:**
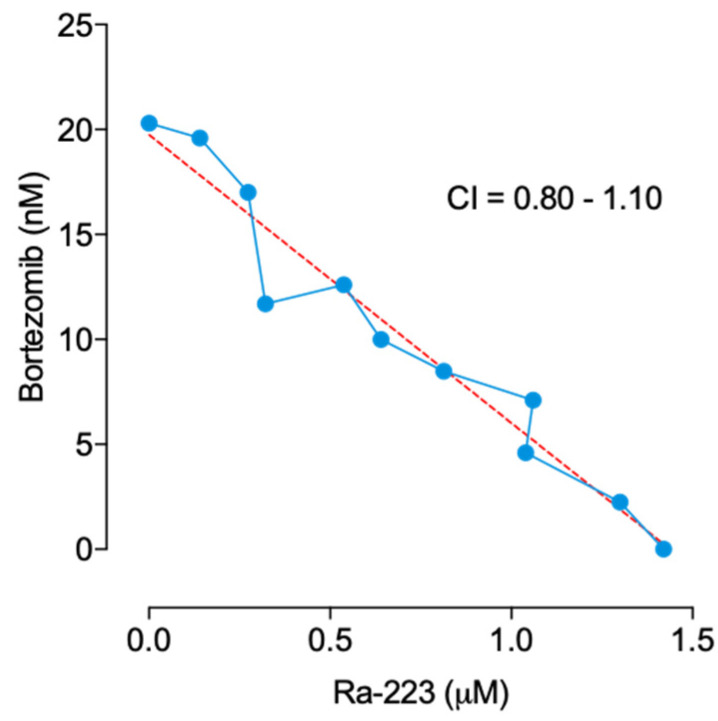
IC_50_ isobologram for the combination effect of radium-223 dichloride (Ra-223) and bortezomib on the proliferation of 5GTM1 MM cells. CI, combination index.

The in vitro activity of 200 and 800 Bq/mL radium-223 and 2.5 and 25 nM bortezomib was investigated in various MM cell lines, including the 5TGM1 mouse MM cell line and the JJN-3, LP-1, MOLP-8, RPMI-8226, and OPM-2 human cancer cell lines. In the tested cell lines, the treatment with radium-223 at 800 Bq/mL for 4 days resulted in a 16.7–73.6% inhibition of cell proliferation compared to the vehicle-treated cells (*p* < 0.001). In the MOLP-8 cell line, radium-223 showed antiproliferative responses at 200 Bq/mL (22.2% inhibition, *p* < 0.01) (Table 1). Bortezomib inhibited proliferation of all six cell lines tested at 25 nM concentration (91.1–99.5%, *p* < 0.001) and JJN-3 and OPM-2 at 2.5 nM (11.0% inhibition, *p* < 0.001 and 18.1%, *p* < 0.05, respectively). Combination of radium-223 at 200 Bq/mL with bortezomib at 2.5 nM showed increased inhibition of cell proliferation in 5TGM1, JJN-3, LP-1, and OPM-2 when compared to the corresponding monotherapy group (Table 1). This in vitro antiproliferative combination effect was further assessed by combination indexes and isobolograms of IC_50_ in 5TGM1 cells. The interaction of radium-223 with bortezomib had an additive inhibitory effect on cell proliferation with combination indexes ranging from 0.80 to 1.10 (Figure 1).

### 2.2. Radium-223 and Bortezomib Combination Treatment Is Well Tolerated and Delays Disease Progression in the 5TGM1 Mouse Myeloma Model

**Figure 2:**
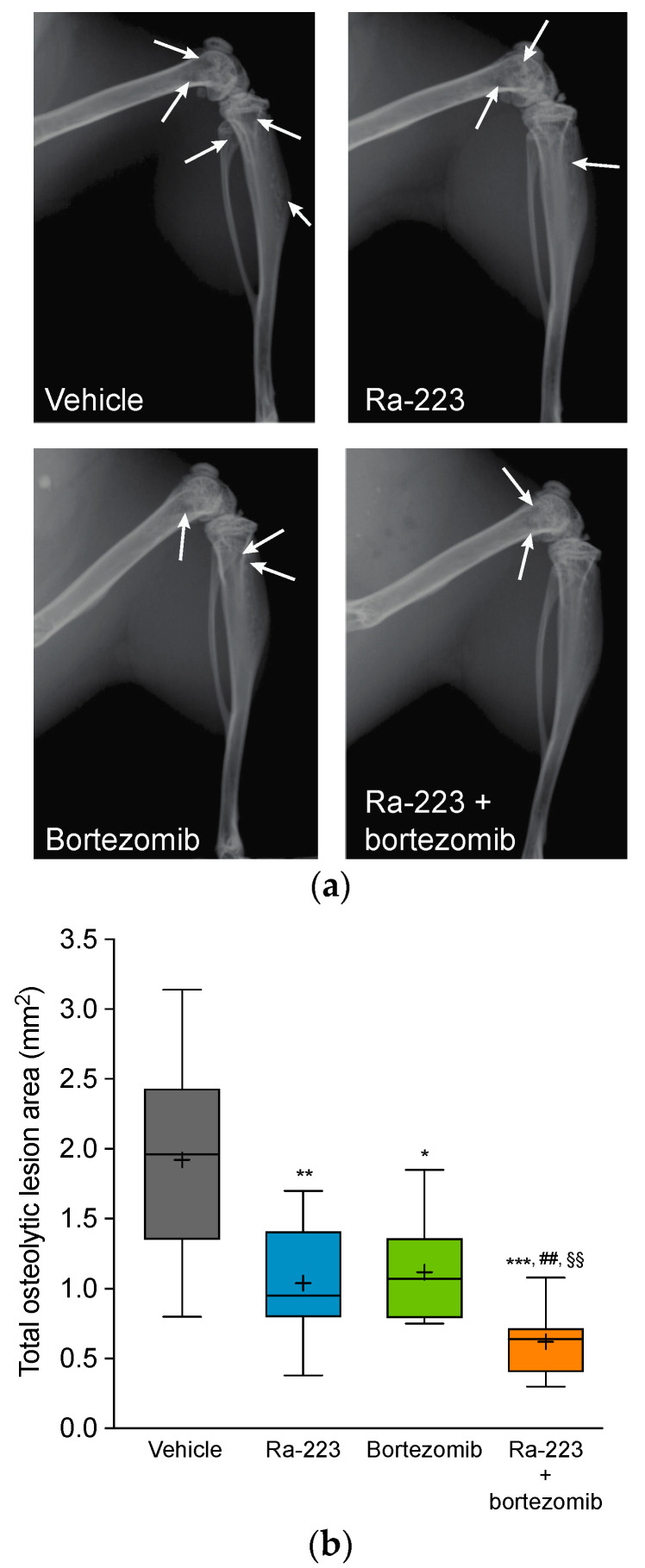
Radium-223 and bortezomib in mono- or combination treatment inhibit tumor-induced bone destruction in the 5TGM1 myeloma mouse model with established bone disease. Mice were treated with vehicle, radium-223 dichloride (Ra-223; 330 kBq/kg, single i.v. injection), or bortezomib (1 mg/kg, i.p., twice a week). (**a**) Representative X-ray images of mice hind limbs analyzed for osteolytic lesions at sacrifice on study day 35. One animal shown from each group. Osteolytic areas are indicated by white arrows. (**b**) Total osteolytic lesion area in bone determined from both hind limbs at sacrifice using X-ray images and image analysis software. Horizontal lines represent the 5th, 25th, 50th, 75th, and 95th percentiles and crosses indicate mean values. * *p* < 0.05, ** *p* < 0.01, and *** *p* < 0.001 compared to vehicle; ## *p* < 0.01 compared to radium-223 monotherapy; §§ *p* < 0.01 compared to bortezomib monotherapy.

The in vivo effect of radium-223 on myeloma bone disease as a single agent or in combination with bortezomib was investigated using the systemic, syngeneic 5TGM1 MM mouse model. Treatment was initiated 26 days after tumor cell inoculation in animals with elevated IgG2b levels, indicative of systemic MM disease. The development of tumor-induced osteolytic bone lesions was inhibited by all therapies compared to vehicle control. Radiographic analysis revealed 41%, 36%, and 66% decreases in the osteolytic lesion area in the radium-223 (*p* < 0.01) and bortezomib (*p* < 0.05) monotherapy groups and the combination therapy group (*p* < 0.001), respectively, as compared to vehicle. Combination treatment decreased osteolytic lesions more effectively than either of the monotherapies alone (*p* < 0.01 for both) (Figure 2).

**Figure 3:**
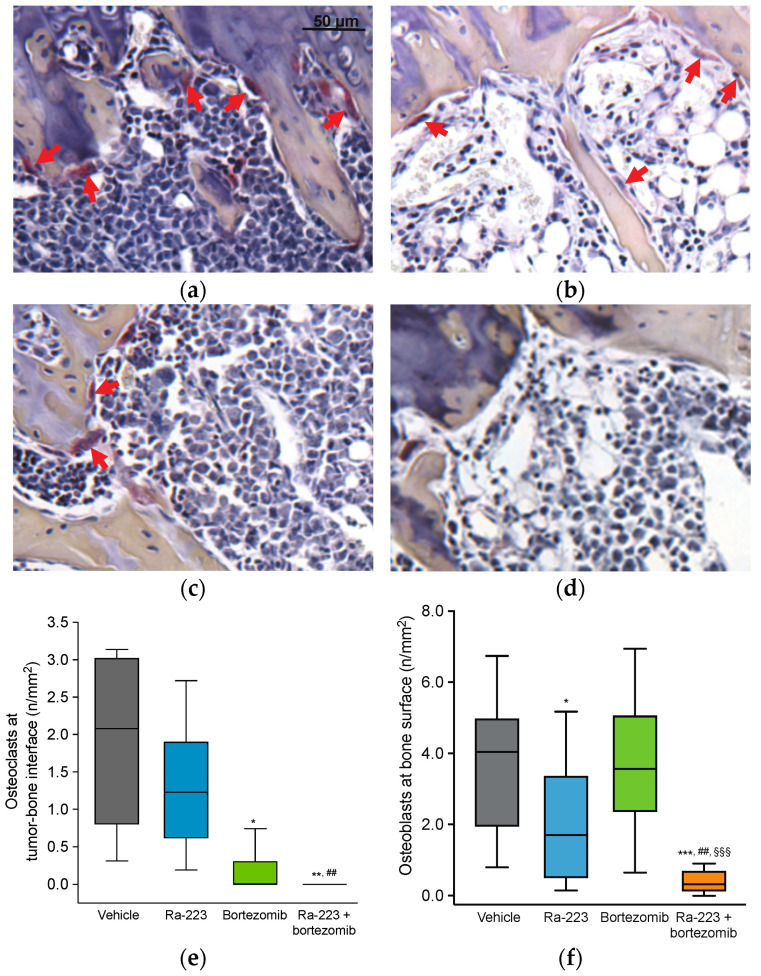
Effect of radium-223 and bortezomib in mono- or combination treatment on the number of osteoclasts and osteoblasts in the 5TGM1 myeloma mouse model. Representative histology images of tartrate-resistant acid phosphatase (TRAP) stained sections after treatment with (**a**) vehicle control, (**b**) radium-223 dichloride (Ra-223; 330 kBq/kg, single i.v. injection), (**c**) bortezomib (1 mg/kg, i.p., twice a week), or (**d**) their combination. Osteoclasts at the tumor–bone interface are indicated by red arrows. Scale bar indicates 50 μM and is representative for all images. (**e**) Number of osteoclasts at the tumor–bone interface. (**f**) Number of osteoblasts at bone surface. * *p* < 0.05, ** *p* < 0.01 and *** *p* < 0.001 compared to vehicle; ## *p* < 0.01 compared to radium-223 monotherapy; §§§ *p* < 0.001 compared to bortezomib monotherapy.

Histological examination of bone sections revealed that bortezomib monotherapy decreased the number of osteoclasts at the bone–tumor surface (*p* < 0.05 vs. vehicle). An enhanced effect was observed with combination therapy resulting in almost complete eradication of osteoclasts (*p* < 0.01 vs. vehicle and radium-223 monotherapy group) (Figure 3). The number of osteoblasts at the bone surface was also decreased by the combination treatment (*p* < 0.001 vs. vehicle), whereas radium-223 monotherapy resulted in a tendency of lower number of osteoblasts (*p* < 0.1) (Figure 3f).

**Figure 4:**
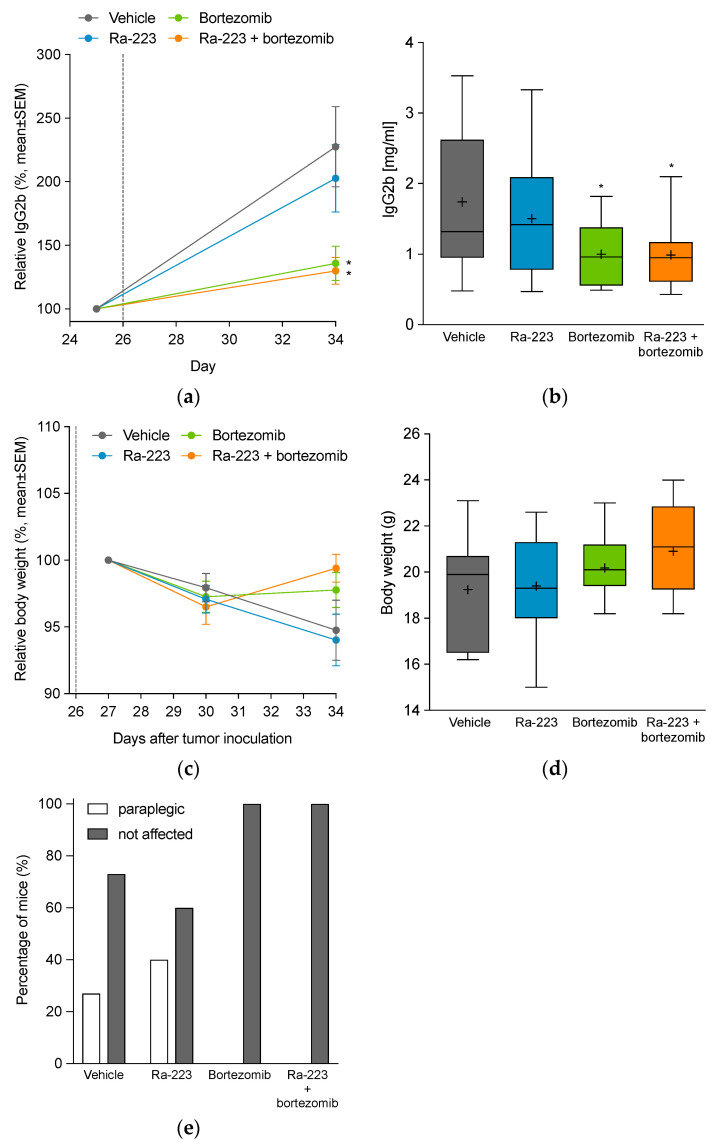
Effect of radium-223 and bortezomib in mono- or combination treatment on serum IgG2b levels and body weight in the 5TGM1 myeloma mouse model. Mice were treated with vehicle, radium-223 dichloride (Ra-223; 330 kBq/kg, single i.v. injection), or bortezomib (1 mg/kg, i.p., twice a week) as single agents or in combination. (**a**) Relative serum IgG2b paraprotein levels during the study (change from day 25 to day 34). Treatment start is indicated with a dotted line. (**b**) Serum IgG2b levels (absolute values) at the end of the study on day 34. (**c**) Relative mouse body weights during the study (change from day 27 to day 34). Treatment start (day 26) is indicated with a dotted line. (**d**) Mouse body weights (absolute values) at the end of the study on day 34. (**e**) Number of mice with paraplegia during the study. Horizontal lines in (**b**,**d**) represent the 5th, 25th, 50th, 75th, and 95th percentiles; crosses indicate mean values. * *p* < 0.05 compared to vehicle.

Disease progression in the 5TGM1 MM mouse model is typically accompanied by increased levels of serum paraprotein IgG2b [23,24,25], reflecting the whole-body tumor burden of mice. In the vehicle and radium-223 monotherapy groups, serum IgG2b showed 1.7- and 2.0-fold increases compared with baseline (on day 25), respectively. Treatment with bortezomib alone or in combination with radium-223 resulted in noticeably lower serum IgG2b levels, with 1.4- and 1.3-fold increases (*p* < 0.05 vs. vehicle for both) compared with baseline (on day 25), respectively, indicative of decreased myeloma burden (Figure 4a,b).

Substantial weight loss and paraplegia are indicators of disease progression in the 5TGM1 MM mouse model. In the vehicle, radium-223, and bortezomib monotherapy groups, mice experienced weight losses of 5.3%, 6.0%, and 2.2%, respectively. Remarkably, no body weight loss was observed in the group treated with the combination of bortezomib and radium-223 (Figure 4c,d). Paraplegia occurred in 27% and 43% of mice in the vehicle and radium-223 monotherapy groups, respectively, while bortezomib as a single agent or in combination with radium-223 completely prevented tumor-associated paraplegia (Figure 4e). No macroscopic findings were noticed in any of the organs including kidney and heart at autopsy. Furthermore, kidney weights did not differ between the treatment groups. Taken together, these results indicated that combination therapy lowered tumor burden, restored body weight, and prevented paraplegia, indicating delayed disease progression and high tolerability in the 5TGM1 MM mouse model.

### 2.3. Combination Effect of Radium-223 and Bortezomib Is Not Altered by Dexamethasone

**Figure 5:**
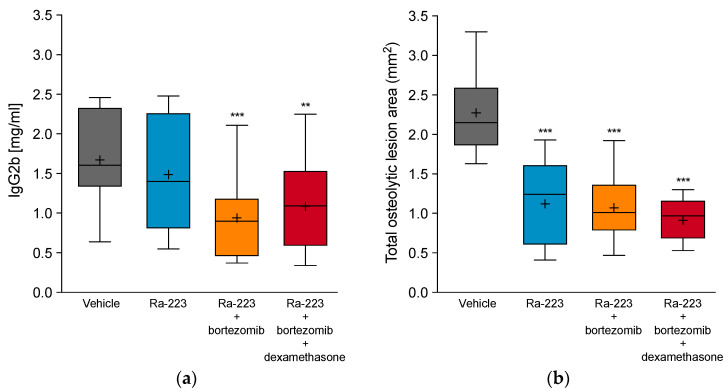
Effect of radium-223, bortezomib, and dexamethasone combination treatment on serum IgG2b levels and osteolytic lesion area in the 5TGM1 myeloma mouse model. Mice were treated with vehicle, radium-223 dichloride (Ra-223; 330 kBq/kg, single i.v. injection), and a combination of radium-223 (330 kBq/kg, single i.v. injection) with either bortezomib (1 mg/kg, i.p., twice a week) or bortezomib and dexamethasone (1 mg/kg, i.p., QD, 5 days on/2 days off). (**a**) Serum IgG2b levels (absolute values) at the end of the study on day 34. (**b**) Total osteolytic lesion area in bone determined from both hind limbs at sacrifice using X-ray images and image analysis software. Horizontal lines represent the 5th, 25th, 50th, 75th, and 95th percentiles and crosses indicate mean values. ** *p* < 0.01 and *** *p* < 0.001 compared to vehicle.

Given the frequent use of bortezomib in combination with dexamethasone in the clinical setting, we next investigated whether dexamethasone had an impact on the in vivo effects of radium-223 and bortezomib combination therapy in the 5TGM1 MM mouse model. This triple combination treatment resulted in decreased serum IgG2b levels and decreased tumor-induced bone destruction as compared to vehicle (Figure 5). Observed effects were of the same magnitude as for the combination of radium-223 and bortezomib, indicating that dexamethasone treatment did not interfere with the combination treatment of the other two drugs. The triple combination treatment was also well tolerated with body weight losses of less than 10%.

### 2.4. Radium-223 and Bortezomib Combination Treatment Results in Tumor Lesion Necrosis

**Figure 6:**
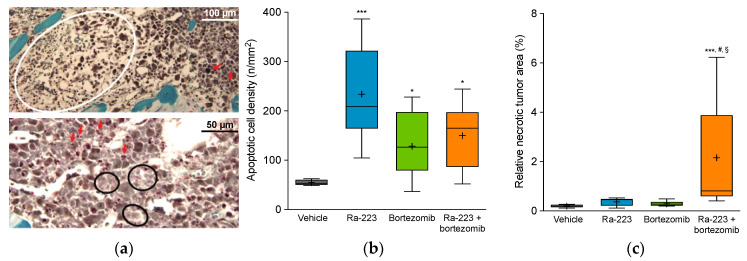
Effect of radium-223 and bortezomib in mono- or combination treatment on apoptosis, necrosis, and fibrosis in tumor-bearing tibias. Mice were treated with vehicle, radium-223 dichloride (Ra-223; 330 kBq/kg, single i.v. injection), or bortezomib (1 mg/kg, i.p., twice a week) as single agents or in combination. (**a**) Representative histological images (400x magnification) of MGT-stained sections of mouse tibias after treatment with radium-223 in combination with bortezomib. Arrows indicate apoptotic cells, the white circle indicates fibrotic area (i.e., evidence of past necrosis, lymphocytes, fibroblasts, and remaining tumor cells), and black circles indicate necrotic area. Scalebars indicate 100 μm and 50 μm in the upper and lower images, respectively. (**b**) Number of apoptotic cells relative to tumor area (n/mm^2^) analyzed from TUNEL-stained sections. (**c**) Necrotic tumor area relative to total tumor area analyzed from TUNEL-stained sections. Horizontal lines in (**b**,**c**) represent the 5th, 25th, 50th, 75th, and 95th percentiles and crosses indicate mean values. * *p* < 0.05 and *** *p* < 0.001 compared to vehicle; # *p* < 0.05 compared to radium-223 monotherapy; § *p* < 0.05 compared to bortezomib monotherapy.

Microscopic evaluation of tumor-bearing tibiae revealed the presence of apoptotic cells and necrotic tumor areas, as well as signs of fibrosis (i.e., evidence of past necrosis, lymphocytes, fibroblasts, and few remaining tumor cells), suggesting a multiform and complex phenotype (Figure 6a). Increased numbers of apoptotic cells were detected in all treatment groups compared to vehicle, with 4.3-, 2.4-, and 2.8-fold increases for radium-223 (*p* < 0.001), bortezomib (*p* < 0.05), and combination therapy (*p* < 0.05) groups, respectively (Figure 6b). Marked induction of tumor necrosis was detected in the combination therapy group with an 11.2-fold increase as compared to vehicle (*p* < 0.001), whereas, in the radium-223 or bortezomib monotherapy groups, 1.9- and 1.5-fold increases, respectively, in necrotic tumor foci were observed (Figure 6c). Importantly, the induction of tumor necrosis with the combination treatment was synergistic (*p* < 0.05).

### 2.5. Radium-223 and Bortezomib Combination Treatment Results in Higher Radium-223 Incorporation into Bone

**Figure 7:**
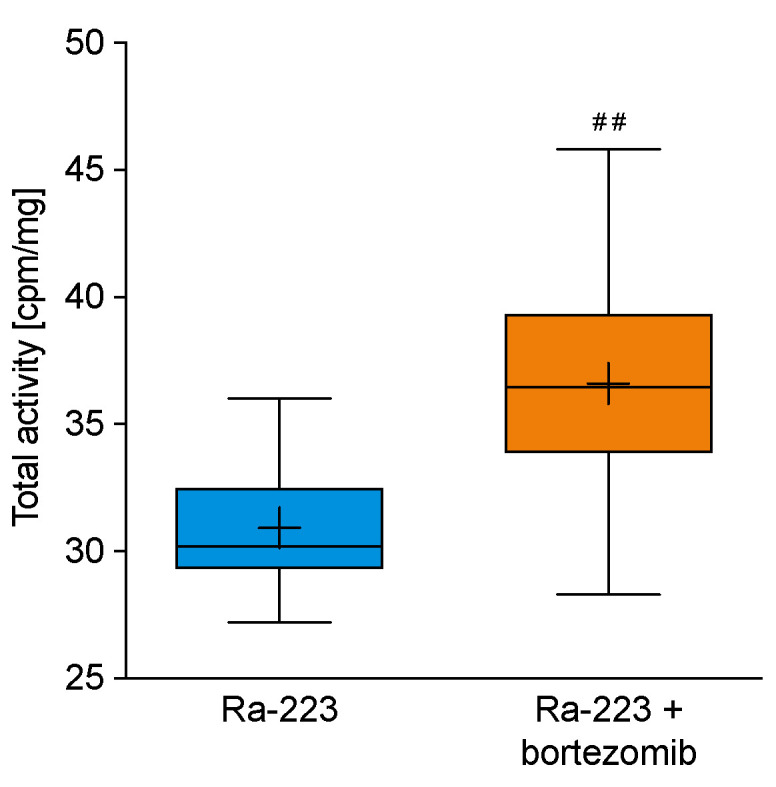
Radium-223 incorporation into the bone matrix. The mice bearing 5TGM1 MM were treated with radium-223 dichloride (Ra-223; 330 kBq/kg, single i.v. injection) as a single agent or in combination with bortezomib (1 mg/kg, i.p., twice a week). Total activity (cpm/mg) of hind limbs at sacrifice on day 35. Horizontal lines in box plots represent the 5th, 25th, 50th, 75th, and 95th percentiles and crosses indicate mean values. ## *p* < 0.01 compared to radium-223 monotherapy.

To elucidate the mechanism behind the beneficial effects of the combination treatment with radium-223 and bortezomib in the 5TGM1 MM disease model, the incorporation of radium-223 into the bone matrix was determined by total activity measurements of the hind limbs. The radioactivity of the bone matrix at sacrifice was 30.9 cpm/mg in the radium-223 monotherapy and 36.6. cpm/mg in the combination therapy groups, indicating increased radium-223 incorporation (*p* < 0.01) with combination therapy (Figure 7).

## 3. Discussion

Myeloma bone disease is a devastating condition affecting as many as 80% of MM patients. This condition is characterized by destructive osteolytic lesions causing bone pain, fractures, and hypercalcemia, and it is usually incurable [2,3,4]. Physiological bone remodeling is coordinated by the interactions between the bone matrix, osteocytes, osteoclasts, osteoblasts, and immune cells. In myeloma bone disease, this intricate interaction is significantly disrupted. In particular, increased osteoclast activity and suppressed osteoblast function deregulate bone turnover, leading to bone loss and skeletal-related events [26].

In recent years, remarkable progress has been made in the understanding of MM disease mechanisms, and this has led to new, more targeted treatment options. These agents represent different modes of action, including first- and second-generation proteasome inhibitors, immunomodulators, and monoclonal antibodies binding to CD38 [27,28,29,30,31,32]. This diversity has also allowed combination regimens to be explored and taken into routine clinical practice [29,30,31,32,33,34,35]. In second-line therapy, combination treatment is chosen by default, with the choice of agents depending on prior treatment or on whether the MM status is refractory or relapsed [29,30,31,32,34].

Bortezomib is a first-generation proteasome inhibitor, and it has played an important role in MM treatment since its approval by the FDA and EMA in the early 2000s, considerably improving the outcome of MM patients [6,36,37]. However, the use of bortezomib is limited by toxicities and resistance even after good initial response to the drug [6,8,38]. Recently, novel second-generation proteasome inhibitors, such as carfilzomib and ixazomib, have been developed to overcome bortezomib resistance [39,40,41]. In preclinical studies, bortezomib and the new proteasome inhibitors have been shown to inhibit osteoclast differentiation, mainly through blockade of the RANKL signaling pathway in osteoclast progenitors. However, the more significant effect on bone remodeling by this class of drugs is their capacity to stimulate osteoblast differentiation [39,40,41]. Clinical evidence of increased bone formation markers and decreased bone resorption markers highlight the crucial role of proteasome inhibitors in increasing osteoblast and suppressing osteoclast function, respectively [39,40].

Radium-223 is an alpha particle (α)-emitting, bone-seeking calcium mimetic that binds to hydroxyapatite in bone and accumulates in areas of increased bone turnover, thus delivering cytotoxic radiation to the sites of bone metastases [42,43,44]. Radium-223 is a self-targeting TAT that can be incorporated into bone both passively due to its physical properties and actively via osteoblast function. The primary advantage of α emitters over other types of radioactive sources is their very high linear energy transfer and the resulting highly cytotoxic effects, as well as their high specificity due to their very short path length (50–100 µm) [13,43]. In bone metastasis models, radium-223 has been shown to exhibit a dual-targeting mode of action that inhibits disease progression by inducing tumor cell death and by suppressing disease-promoting osteoclasts and osteoblasts in the tumor microenvironment [17].

The primary aim of this study was to evaluate the efficacy of radium-223 in combination with bortezomib. In particular, we wanted to investigate whether the efficacy of radium-223 would be impaired due to the disease-related inhibition of osteoblasts, which could result in decreased uptake of radium-223 into newly formed bone [18]. We hypothesized that the combination of different modes of action, namely proteasome inhibition by bortezomib, resulting in the reconstitution of osteoblast function, and alpha particle emission by radium-223, targeting areas of increased bone turnover, could constitute a novel, improved therapeutic strategy for myeloma bone disease.

In our study, the combination of bortezomib with radium-223 inhibited the proliferation of MM cell lines more efficiently than either of the monotherapies alone. Due to the different modes of action of these compounds, synergistic or antagonistic in vitro effects were not anticipated. As expected, the interaction of bortezomib with radium-223 proved to be additive in the 5TGM1 MM cell line, supporting further evaluation in the in vivo setting.

For the in vivo combination studies, we used the 5TGM1 MM mouse model that mimics the features of active MM in humans, including prominent osteolytic lesions in bone [22,23,24,25]. Despite the fact that this model is known for its aggressive disease progression [23], the combination therapy with radium-223 and bortezomib was able to induce a pronounced reduction of the osteolytic area in a treatment period of only 9 days. Moreover, the number of disease-driving osteoclasts at the tumor–bone interface was almost completely eradicated, indicating enhanced inhibition of tumor-induced skeletal events upon combination therapy. The number of osteoblasts, however, was decreased in both radium-223 monotherapy and the combination treatment group. The half-life of radium-223 is only 11.4 days. Consequently, the effect of radium-223 would be temporary, and therefore, new osteoblasts would continue to differentiate from mesenchymal stem cells. This assumption is supported by our previous findings in a mouse model of breast cancer bone metastasis, where no changes in the number of osteoblasts were observed 10 days after the radium-223 treatment [18].

Although radium-223 monotherapy showed no effect on the whole-body tumor burden of the mice, radium-223 in combination with bortezomib resulted in a marked reduction of the tumor burden when compared to the vehicle control group. Based on histology, the combination treatment increased necrotic area in the tumor more than either of the monotherapies alone. Importantly, this effect was shown to be synergistic. Furthermore, disease-induced paraplegia was completely prevented both in the bortezomib monotherapy and in the combination treatment group. Moreover, body weight change provides additional evidence for the efficacy and tolerability of the combination treatment. In animal models characterized by osteolytic bone disease-related body weight loss, the efficacy of radium-223 treatment has translated into restoration or gain of body weight [17]. In line with this, our results demonstrated that the radium-223 and bortezomib combination treatment restored body weight, thus, confirming the efficacy and safety of the treatment. In order to assess the influence of these treatments on survival, the effect on spleen weight and the bone marrow plasmacytosis should be evaluated, in addition to the parameters measured in this study [45].

In the clinical setting, bortezomib has been applied frequently in combination with dexamethasone, although its use has become less widespread since the emergence of novel double and triple combination treatments [29]. Dexamethasone has pronounced effects on bone, including osteoporosis and osteonecrosis [46,47], and therefore, we also evaluated the triple combination of radium-223, bortezomib, and dexamethasone in the present study. Our results indicate that the triple combination with dexamethasone was well tolerated and that dexamethasone did not affect the efficacy of the radium-223 and bortezomib combination treatment. It should be noted, however, that the treatment period in this triple combination experiment was not ideal, and therefore, it should be repeated using a similar study design as in the radium-223 and bortezomib combination experiment. More importantly, new and clinically more relevant combinations should be evaluated using this aggressive model of myeloma bone disease.

Previous studies have demonstrated that radium-223 causes potentially cytotoxic, difficult-to-repair DSBs in cancer cells metastasized to bone and that these effects are detectable as early as 24 h post-treatment [13,17,18]. In our study, the consequences of DSB induction, namely increased tumor cell death, necrosis and fibrosis, were detected nine days after the initiation of the combination therapy. Increased necrosis in combination with the modest increase in the number of apoptotic cells suggests that the damaged cancer cells in the radium-223 and bortezomib combination treatment group may, in fact, have died before analysis, which was performed at sacrifice, nine days after the initiation of treatment. Observed fibrosis in the combination treatment group further supported this hypothesis.

Taken together, the combination of bortezomib with radium-223 demonstrated additive antitumor efficacy both in vitro and in vivo. Two mechanisms may contribute to the beneficial combination effect. Regarding the observed in vitro effects, bortezomib may enhance the consequences of the radium-223-induced induction of DSBs. Bortezomib is known to be capable of sensitizing cancer cells to both chemo- and radiotherapy by disrupting de novo and acquired resistance [48,49] and via downregulation of proteins involved in DNA repair [50,51]. Regarding the in vivo situation, the observed increase in radium-223 incorporation into bone upon combination treatment may also play an important role. Bortezomib and other proteasome inhibitors have been shown to restore the impaired osteoblast activity typically observed in MM [9,10,11], potentially resulting in an increased osteoblast-mediated radium-223 incorporation.

A Phase 1b/2 clinical trial had been initiated to determine the optimal dose of radium-223 dichloride in combination with bortezomib/dexamethasone for the Phase 2 portion of the study (ClinicalTrials.gov, accessed on 31 March 2021, Identifier: NCT02928029). Due to the changes in standard of care and the slow recruitment of participants, the study was terminated before the maximum tolerated dose had been reached. The potential benefit of radium-223 in multiple myeloma should be further explored in preclinical studies conducted with new proteasome inhibitor-containing combinations recently approved.

## 4. Materials and Methods

### 4.1. Cells and Compounds

The 5TGM1 mouse MM cell line and the JJN-3, LP-1, L-363, MOLP-8, RPMI-8226, and OPM-2 human hematological cancer cell lines were cultivated according to supplier protocols (Supplementary Table S1) in a humidified atmosphere containing 5% CO_2_ and passaged less than 10 times during the study. All cell lines were authenticated by the provider (Supplementary Table S1), except 5TGM1 mouse MM cells, for which authentication was not possible due to lack of reference authentication data. The myeloma cell identity of 5TGM1 cells was confirmed by the secretion of the MM-specific IgG2b paraprotein and tumor formation in the specific C57Bl/KalwRij syngeneic mouse strain. Cell lines were tested to be free from mycoplasma contamination using MycoAlert (Lonza, Cologne, Germany).

Radium-223 dichloride was synthesized at Algeta ASA (Oslo, Norway) as previously described [52], with an activity concentration of 60 kBq/mL. Bortezomib was obtained from LC Laboratories (Woburn, MA, USA) and prepared fresh for every dosing (0.2 mg/mL). Dexamethasone 21-phosphate disodium salt was obtained from Sigma-Aldrich (St Louis, MO, USA), and fresh aliquots (0.2 mg/mL, stored at −20 °C) were thawed before every dosing.

### 4.2. In Vitro Proliferation Assays

The effect of radium-223 and bortezomib on cell proliferation was evaluated in 5TGM1, JJN-3, LP-1, L-363, MOLP-8, RPMI-8226, and OPM-2 cells using the CellTiter-Glo cell viability assay (Promega, Madison, WI, USA). The inhibition of cell proliferation by radium-223 (200 or 800 Bq/mL) and/or bortezomib (2.5 or 25 nM) was measured on days 1, 2, 3, and 5. Radium-223 was added on day 0 as described in detail in the Supplementary Methods.

The in vitro antiproliferative activity of radium-223 (20–2560 Bq/mL; equaling 0.3–40 nM) in combination with bortezomib (0.3–40 nM) was assessed in 5TGM1 cells by determination of combination indexes [4]. Cells were treated with a single compound or a combination of nine fixed compound ratios for four days, and viability was measured using Cell Titer-Glo. IC_50_ values were calculated from triplicate values for each individual combination data point, and the respective isobologram was generated. Combination indexes were calculated according to the median-effect model [53]. A CI of ≤ 0.8 was defined as more than additive (i.e., synergistic) interaction, 0.8 < CI < 1.2 indicated an additive effect, and a CI of ≥1.2 was defined as antagonistic interaction.

### 4.3. Animals

All animal experiments were approved by the Animal Experiment Board of Finland (license numbers: ESAVI-06057-04.10.03-2011 and ESAVI-2077-04-10 07-2014) and performed according to the guidelines of the European Union directive 2010/63/EU. Experiments were initiated after an acclimatization period of at least 5 days. Mice were housed in groups of 5, and food and water were available ad libitum.

### 4.4. In Vivo Studies in the Syngeneic 5TGM1 Mouse Multiple Myeloma Model

The in vivo tolerability and antitumor efficacy of radium-223 and bortezomib as mono- or combination therapy were evaluated in the systemic, syngeneic 5TGM1 MM mouse model. In addition, a triple combination study with radium-223, bortezomib, and dexamethasone was evaluated in the same model. In 2015, the National Institute of Standards and Technology (NIST) revised the primary standardization for radium-223 [54]; hence, the numerical value of the radioactivity dose administered to mice was increased by approx. 10%, from 300 to 330 Bq/kg. This did not reflect a real change in the actual amount of radioactivity given to the mice.

5TGM1 mouse MM cells were inoculated intravenously into the tail vein of female C57BL/KaLwRij specific pathogen-free (SPF) mice (14–22 g for 7–9 weeks in the combination study with radium-223 and bortezomib or 14–20 g for 8–9 weeks in the triple combination study with radium-223, bortezomib, and dexamethasone, respectively; Envigo, Huntingdon, UK). Within 4–6 weeks, the mice developed a bone disease with typical characteristics of active human MM, including secretion of IgG2b paraprotein and pronounced osteolytic lesions in the bone [23,24,25,55]. Allocation to treatment groups (*n* = 15 per group) was performed using a stratification procedure based on the serum IgG2b level (indicative of systemic MM disease) on day 25. Treatment was initiated on day 26.

For combination therapy experiments with radium-223 and bortezomib, mice were treated with radium-223 (330 kBq/kg, single i.v. injection) or bortezomib (1 mg/kg, i.p., twice a week) as single agents or in combination. For the triple combination experiment (radium-223, bortezomib, and dexamethasone), mice were additionally treated with 1 mg/kg dexamethasone (i.p., QD, 5 days on/2 days off). Mice were sacrificed on day 35 after cancer cell inoculation, or earlier if they became moribund. Tissue samples from hind limbs (left and right tibiae and femora) were collected for histology. Histological stainings and histomorphometry were performed, and osteoclasts, osteoblasts, and apoptotic and necrotic cells were determined as explained in the Supplementary Methods. Blood samples were collected from the saphenous vein before the inoculation of cancer cells (day -1) and on days 25 (21 in the triple combination therapy experiments) and 34 after cancer cell inoculation (or when sacrificed). The development of myeloma-induced osteolytic lesions in bone and tumor growth were assessed using X-ray radiography and by measuring tumor paraprotein IgG2b levels (mouse IgG2b ELISA quantitation kit, Bethyl Laboratories Inc., Montgomery, TX, USA) in serum, respectively. Animal body weight was monitored as a measure of disease progression and possible treatment-related toxicity. Details of the in vivo experiments are described in Supplementary Methods.

### 4.5. Statistical Analyses

Statistical analyses were performed using R (version 3.1.0 or newer, www.r-project.org, accessed on 31 March 2021). The end-point parameters including relative biomarker values were analyzed using one-way ANOVA followed by model contrasts or using Kruskal–Wallis test followed by Dunn’s post hoc test. The interaction and main effects of radium-223 and bortezomib were also analyzed using two-way ANOVA model with terms of presence/absence of the two compounds and their interaction. Statistically significant interaction term of the model implies either synergistic or antagonistic effect between the two compounds, and the type of the effect can be determined using means of the four groups. Weight curves were analyzed using mixed models as relative values compared to baseline and compared using model contrasts. Paraplegia was analyzed using Fisher’s exact test. All the obtained *p*-values were adjusted for multiple comparisons.

## 5. Conclusions

According to our studies, radium-223 and bortezomib combination treatment demonstrates additive efficacy both in vitro and in vivo. In particular, the inhibition of multiple myeloma growth and myeloma-induced bone destruction is more prominent in the combination treatment group compared to either of the monotherapies alone. Taken together, the data presented here suggest that the combination of radium-223 and bortezomib is a promising novel therapy in MM and provide a rationale for further studies in the clinical setting.

## Figures and Tables

**Table 1:** Inhibitory effect of radium-223, bortezomib, and radium-223 in combination with bortezomib on cell proliferation in various multiple myeloma cell lines.

Cell Line	Radium-223 800 Bq/mL	Radium-223 200 Bq/mL	Bortezomib 25 nM	Bortezomib 2.5 nM	Ra-223, 200 Bq/mL Bortezomib, 2.5 nM
5TGM1	16.7% ***	0%	99.3% ***	4.1%	9.5% ***^, ###, §§§^
JJN-3	26.5% ***	0%	99.5% ***	11.0% ***	29.1% ***^, ###, §§§^
LP-1	20.0% ***	0%	99.3% ***	6.2%	11.6% ***^, ###, §§§^
MOLP-8	73.6% ***	22.2% **	93.7% ***	19.0%	41.3% ***^, §§^
RPMI-8226	46.8% ***	13.5%	91.1% ***	0%	13.1%
OPM-2	52.3% ***	13.3%	99.5% ***	18.1% *	31.0% ***^, ##, §§^

* *p* < 0.05, ** *p* < 0.01, and *** *p* < 0.001 compared to vehicle; ## *p* < 0.01 and ### *p* < 0.001 compared to radium-223 monotherapy at 200 Bq/mL; §§ *p* < 0.01 and §§§ *p* < 0.001 compared to bortezomib monotherapy at 2.5 nM; Ra-223, radium-223.

## Data Availability

The data presented in this study are available on request from the corresponding author.

## Supplementary Materials

The following are available online at https://www.mdpi.com/article/10.3390/ijms22115570/s1.
