# Supplementary material for: Additive Benefits of Radium-223 Dichloride and Bortezomib Combination in a Systemic Multiple Myeloma Mouse Model
Source: Int J Mol Sci. 2021 May 25;22(11):5570. doi: 10.3390/ijms22115570 (PMC8197539; doi:10.3390/ijms22115570)
Supplement: Supplementary file 1 [file ijms-22-05570-s001.zip › ijms-1185661-supplementary.pdf]

Supplementary information for:

Additive benefits of radium-223 dichloride and bortezomib combination in a systemic multiple myeloma mouse model

Mari I Suominen, Jenni Mäki-Jouppila, Anna Huhtinen, Birgitta Sjöholm, Jukka P Rissanen, Anniina Luostarinen, Katja M Fagerlund, Esa Alhoniemi, Gerhard Siemeister, Dominik Mumberg, Sanna-Maria Käkönen, Arne Scholz

Supplementary methods

In vitro proliferation

The anti-proliferative activity of radium-223 was measured using the CellTiter-Glo® (CTG) cell viability assay (Promega, Madison, WI, USA). 5TGM1, JN-3, LP-1, L-363, MOLP-8, RPMI-8226 and OPM-2 cells were plated on 96-well plates in 100 µl of growth medium. Radium-223 (200 or 800 Bq/ml), bortezomib (2.5 or 25 nM) and the reference inhibitor doxorubicin (0.1 µM) were added on day 0, resulting in a total volume of 200 µl. DMSO and sodium citrate buffer (28 mM) were used as vehicles. On days 1, 2, 3, and 5, CTG reagent was added into the wells and incubated for 30 min at room temperature. Luminescence was measured using the Victor2™ Multilabel Counter (PerkinElmer, Waltham, MA, USA).

In vivo studies in the 5TGM1 mouse multiple myeloma model

The *in vivo* antitumor efficacy and tolerability of radium-223 and bortezomib as mono- or combination therapy and were evaluated in the syngeneic 5TGM1 mouse MM model. In addition, a triple combination study with radium-223, bortezomib and dexamethasone was evaluated in the same model. On day 0, female C57BL/KaLwRij-specific pathogen free (SPF) mice (7-9 weeks, 14-22 g or 8-9 weeks, 14-20 g in the combination study with radium-223 and bortezomib or in the triple combination study with radium-223, bortezomib and dexamethasone, respectively, Envigo, Huntingdon, UK). were inoculated into the tail vein with $2x10^6$ 5TGM1 cells suspended in 100 µl of phosphate buffered saline (PBS). Viability of the cells was determined before and after the inoculation. When serum IgG2b paraprotein secretion had started and pronounced osteolytic lesions had developed, mice were allocated into treatment groups (n = 15 or n = 10-13 in the combination study with radium-223 and bortezomib or in the triple combination study, respectively) by a randomization procedure based on serum IgG2b levels on day 25 or 21 in the combination study with radium-223 and bortezomib or in the triple combination study, respectively. In the radium-223 and bortezomib combination therapy study, treatment with vehicle (vehicle 1: 28 mM citrate buffer, i.v., single dose on day 26; vehicle 2: 0.8% EtOH in 0.9% NaCl, i.p., twice a week),

radium-223 (330 kBq/kg, i.v., single dose on day 26), and bortezomib (1 mg/kg, i.p., twice a week) as single agents or in combination was started on day 26. In the triple combination therapy experiments with radium-223, bortezomib, and dexamethasone, treatment with vehicle (vehicle 1: 28 mM citrate buffer, i.v., single dose on day 22; vehicle 2: PBS, i.p., QD, 5 days on / 2 days off), radium-223 (330 kBq/kg, i.v., single dose on day 22), bortezomib (1 mg/kg, i.p., twice a week), and dexamethasone (1 mg/kg, i.p., QD, 5 days on / 2 days off) as single agents or in combination was started on day 22. Analgesia (0.02 mg/ml buprenorphine in drinking water; Temgesic, Schering-Plough, Kenilworth, NJ, USA) was used for the last 5 days of the study. Mice were sacrificed on day 35 after cancer cell inoculation, or earlier if they became moribund, and imaged immediately by X-ray radiography. Blood samples were collected from the saphenous vein on days -1, 26 (21 in the triple combination study) and 34 after cancer cell inoculation (or when sacrificed). Necropsy was carried out in all animals.

Radiography

Development of the myeloma bone disease was monitored by X-ray radiography immediately at sacrifice. Mice were X-rayed in a prone position with the Faxitron MX-20 D12 Cabinet X-ray System using Faxitron Dicom 3.0 software (Faxitron Corp., Tucson, AZ, USA). At least one radiograph (both hind limbs) per mice was taken on each X-ray occasion (31 kV, 10 s, magnification 2x). The lesion area in the hind limbs was determined from the images with MetaMorph image analysis software (Molecular Devices, San José, CA, USA).

Histology and histomorphometry

Histology and histomorphometry were performed as previously described [1,2]. Tissue samples from hind limbs (left and right tibia and femur) were weighed and collected. Left and right hind limbs were fixed in 4% paraformaldehyde (PFA) or 40% ethanol, respectively, for 2—3 days. Thereafter, the limbs were measured for incorporated radioactivity by gamma-counter before processing to paraffin. The left hind limb was processed to paraffin. Frontal mid-sagittal sections (4 μm thick) were cut with a rotatory

microtome. The sections were stained using Masson-Goldner Trichrome (MGT) staining and scanned with a medical slide scanner. The region of interest (ROI) for bone area measurements was set in a standardized fashion, and the bone area determined by color threshold using the MetaMorph image analysis software (Molecular Devices). Total and trabecular bone areas were determined by drawing separate ROIs and cortical bone area was calculated by subtracting the trabecular area from the total bone area. Tumor area within the trabecular ROI and the whole section were drawn separately. Tumors were verified by microscopy of the same section. Osteoblasts were recognized by morphology and location and counted from the MGT stained sections from two images taken at 100x magnification from each bone. Bone surface length was determined from the same images by drawing with the MetaMorph.

Osteoclasts were counted using tartrate-resistant acid phosphatase (TRAP) staining, based on the presence of red cells containing at least two nuclei and residing on the bone-tumor interface. Images were taken at 25x magnification, and the bone-tumor interface length was measured using MetaMorph software. Apoptosis was analyzed from TUNEL-stained sections by counting stained cells exhibiting apoptotic morphology (200x magnification) and measuring the analyzed tumor area. Necrosis was analyzed by thresholding the red stain on the TUNEL-stained sections. In analyses of apoptosis and necrosis non-tumor objects and empty spaces were excluded from the area.

Supplemental table

Table S1. Cancer cell lines used in the study.

Cell line	Description	Origin	Provider	Culture medium
**5TGM1**	mouse multiple myeloma	bone marrow	Dr. B. Oyajobi, University of Texas Health Science Center at San Antonio (U.S.A.)	IMDM, 15% iFBS, PS
**JJN-3**	human plasma cell leukemia	bone marrow	DSMZ[a]	DMEM, IMDM, 20% iFBS, 2 mM L-Glut, PS
**LP-1**	human multiple myeloma	peripheral blood	DSMZ[a]	IMDM, 20% iFBS, PS
**MOLP-8**	human multiple myeloma	peripheral blood	DSMZ[a]	RPMI 1640, 20% iFBS, 2 mM L-Glut, PS
**RPMI-8226**	human multiple myeloma	peripheral blood	DSMZ[a]	RPMI 1640, 10% iFBS, 2 mM L-Glut, PS
**OPM-2**	human multiple myeloma	peripheral blood	DSMZ[a]	RPMI 1640, 10% iFBS, 2 mM L-Glut, PS

IMDM, Iscove's Modified Dulbecco's Medium; DMEM, Dulbecco's Modified Eagle Medium; iFBS, inactivated fetal bovine serum; L-Glut, L-glutamine; PS, penicillin and streptomycin. Reagents were from Sigma-Aldrich.

[a] DSMZ, Deutsche Sammlung von Mikroorganismen und Zellkulturen / German Collection of Microorganisms and Cell Cultures Research Center (Braunschweig, Germany)

References

17. Suominen, M.I.; Fagerlund, K.M.; Rissanen, J.P.; Konkol, Y.M.; Morko, J.P.; Peng, Z.; Alhoniemi, E.J.; Laine, S.K.; Corey, E.; Mumberg, D.; et al. Radium-223 Inhibits Osseous Prostate Cancer Growth by Dual Targeting of Cancer Cells and Bone Microenvironment in Mouse Models. *Clin. Cancer Res. Off. J. Am. Assoc. Cancer Res.* **2017**, *23*, 4335–4346, doi:10.1158/1078-0432.CCR-16-2955 .

18. Suominen, M.I.; Rissanen, J.P.; Kakonen, R.; Fagerlund, K.M.; Alhoniemi, E.; Mumberg, D.; Ziegelbauer, K.; Halleen, J.M.; Kakonen, S.M.; Scholz, A. Survival benefit with radium-223 dichloride in a mouse model of breast cancer bone metastasis. *J. Natl. Cancer Inst.* **2013**, *105*, 908–916, doi:10.1093/jnci/djt116 .
